# The equity road ahead for financing non-national immunization program vaccines in China: challenges and opportunities from a qualitative study

**DOI:** 10.1186/s12939-024-02282-5

**Published:** 2024-09-27

**Authors:** Mingzhu Jiang, Weixi Jiang, Xuanxuan Yan, Haifeng Ma, Sijuan Zhou, Xiaohua Ying

**Affiliations:** School of Public Health, Fudan University, 130 Dong’an Road, Xuhui District, Shanghai, 200032 China

**Keywords:** Non-NIP vaccines, Vaccination, Financing, Equity, In-depth interview, China

## Abstract

**Background:**

In China, national immunization program (NIP) vaccines benefit from robust financial support and have achieved high coverage. Non-NIP vaccines rely on fragmented funding sources, mostly out-of-pocket payment, and face sub-optimal and inequitable coverage. Sustainable financing needs to be secured for addressing equity in non-NIP vaccine delivery. However, discussion and understanding of this issue remain limited. This study aims to analyze the current situation, comprehensively identify challenges and opportunities in non-NIP vaccine financing, and offer suggestions to enhance vaccine uptake and improve public health.

**Methods:**

Between July and December 2023, we conducted a series of semi-structured, in-person interviews with 55 stakeholders from the Health Bureau, Centers for Disease Control and Prevention, Medical Insurance Bureau, and Finance Bureau across five provinces in China. Participants were selected through stratified sampling, and the interviews mainly included their involvement in non-NIP vaccine financing, challenges faced, and strategies for improvement to enhance financing performance. Informed consent was obtained, and thematic analysis was used to analyze the data.

**Results:**

Non-NIP vaccine financing sources include out-of-pocket payments, government fiscal, health insurance and other external funds. These four channels differ in vaccine types covered, costs, and target populations, each with unique challenges and opportunities. High out-of-pocket costs remain a significant barrier to equitable vaccine uptake, while market competition has lowered the vaccine price and improved accessibility. Local fiscal support for free vaccination programs faces challenges related to sustainability and regional disparity, though governmental commitment to vaccination is growing. Nevertheless, centralized procurement organized by the government has lowered the price and reduced the financial burden. Despite legal restrictions on using basic health insurance for vaccinations and limited commercial insurance options, private medical savings accounts and mutual-aid mechanisms present new opportunities. Although the scope and impact of external support are limited, it has successfully increased awareness and social attention to vaccination.

**Conclusion:**

Relying on individual payments as the main financing channel for non-NIP vaccines is unsustainable and inadequate for ensuring universal vaccine access. A concerted and synergistic approach is essential to ensure sufficient, sustainable resources and enhance public financial management to improve equity in the non-NIP vaccines.

**Supplementary Information:**

The online version contains supplementary material available at 10.1186/s12939-024-02282-5.

## Background

Vaccines are widely acknowledged as the most powerful and cost-effective public health interventions that bring health, economic, and social benefits to people of all ages and communities across the globe [1–3]. Ensuring access to immunization serves as a foundation for the delivery of other essential healthcare services, and is indispensable in advancing Universal Health Coverage (UHC) and achieving the Sustainable Development Goals (SDGs) [4]. Advances in vaccine technologies and the growing number of diseases prevented by vaccines have led to improved well-being [5]. However, the prerequisite for benefiting from these advancements lies in long-term investment for immunization.

In China, vaccines are divided into two categories: National Immunization Program (NIP) and non-NIP vaccines. NIP vaccines are comprehensively funded by the government, ensuring that eligible residents receive them at no personal cost. Non-NIP vaccines are primarily financed out-of-pocket by individuals or guardians of children. Globally, a variety of funding mechanisms are used for these so-called optional or self-paid vaccines, including public health programs, private health insurance, and national immunization subsidies [6]. Additionally, many low-income and lower-middle-income countries benefit from substantial multilateral and external funding support, like the Vaccine Alliance(Gavi), and United Nations Children’s Fund(UNICEF) [7]. As an upper-middle-income country, China’s financial support for non-NIP vaccines is relatively limited and relies on direct out-of-pocket payments. It is noticeable that though the NIP covers 14 types of vaccines, some critically important vaccines recommended by the WHO for inclusion into the NIP of all Member States, such as pneumococcal conjugate vaccine (PCV), human papillomavirus vaccine (HPV), Haemophilus influenzae type b vaccine (Hib), rotavirus vaccines [8], has not been included yet.

Current financing mechanisms for non-NIP vaccines have resulted in vaccination coverage rates that remain far from optimal. A scoping review highlighted that coverage for at least one dose of the Hib vaccine was the highest (54.9–55.9%) while coverage of the other three vaccines (PCV, HPV, rotavirus) was lower than 30% [8]. It is evident that non-NIP vaccine coverage is far lower than the world average and the rate in many developing countries. Besides, non-NIP vaccine coverage shows significant regional disparities in China. Urban areas have much higher rates compared to remote rural regions, resulting in up to a 19-fold difference across provinces [9]. Geographically, the three-dose PCV vaccination rate in 2017 was 2.5% in eastern provinces, compared to just 0.6% and 0.7% in central and western provinces, respectively. 75.8% of children under five in the developed eastern region of Shanghai completed the full Hib vaccination, whereas, in high-burden western regions like Xinjiang, fewer than 3.0% of children were fully vaccinated [10].

In recent years, some provincial or prefectural level governments in China began to subsidize the immunization of selected non-NIP vaccines using the local financial budgets. While these programs increase the non-NIP vaccine coverage in certain regions, they widen the equity gap. Besides, these subsidy programs are not guaranteed by the regular budget, as vaccines are competing for the same financial resources with other public health programs [11], and increasing evidence indicates that resources are being diverted to address other rising healthcare challenges such as aging [12]. Though both Immunization Agenda 2030 (IA2030) and Healthy China 2030 emphasize the need to increase government funding, expand immunization services, and further enhance vaccination coverage and equity across diverse population groups, the financing strategies for securing financing resources remain uncertain. In light of the current challenges in the financing of non-NIP vaccines, an in-depth analysis of the financing schemes for non-NIP vaccines is crucial to identify potential solutions to optimize immunization programs in China. Nevertheless, despite the abundance of research on vaccine financing and international experiences, there was a lack of substantive analysis or qualitative research discussing this issue within the context of China.

This study seeks to elucidate the current status of non-NIP vaccine financing, summarize the associated challenges and opportunities, and propose strategies to enhance financing performance, aiming to optimize the benefits of vaccines to enhance population health and well-being.

## Methods

### Setting

In China, the NIP program was launched in 1978 for newborns and children up to six years old and initially included four vaccines: Bacillus Calmette–Guérin (BCG) vaccine, oral poliovirus vaccine, diphtheria-tetanus-pertussis (DTP) vaccine and measles vaccine. Hepatitis B vaccine was included in 2002. Further expansion occurred in 2008, incorporating hepatitis A, Measles-Mumps-Rubella, Japanese encephalitis, and meningococcal AC vaccines, bringing the total to 14 vaccines covering 15 diseases [13, 14]. However, the NIP has seen no substantial expansion or adjustment in nearly 16 years [15]. The NIP is managed primarily by the National Health Commission (NHC) in collaboration with the National Immunization Advisory Committee (NIAC) and the Ministry of Finance. The NHC is responsible for making evidence-based decisions on the expansion of the NIP, the inclusion of non-NIP vaccines, and the replacement of existing NIP vaccines with new ones. These decisions take into account various factors, such as disease burden, the importance of prevention and control, vaccine cost-effectiveness, program financing, and public confidence [16].

Non-NIP vaccines are those that are not yet included in the NIP and are available to citizens at their own expense and on a voluntary basis. There are over 30 types of non-NIP vaccines and can further be categorized into two main types [8]. The first is alternative non-NIP vaccines, which are designed for diseases already covered by the NIP but differ in characteristics, vaccination procedures and targeting group. For instance, when adults receive a hepatitis B vaccine booster, it is considered as a non-NIP vaccine since it requires out-of-pocket payment. Children under six years old may choose to receive the pentavalent vaccine (DTaP-IPV/Hib), which combines vaccines for diphtheria, pertussis, tetanus, polio, and Hib, which are already included in the NIP except for Hib. The other is supplementary non-NIP vaccines, which are utilized to prevent diseases not yet included in the NIP, such as the PCV, rotavirus vaccine, flu vaccine, and HPV vaccine. Additionally, COVID-19 vaccines in China are used under emergency authorization for public health. As such, they are not within the scope of this study.

**Fig. 1:**
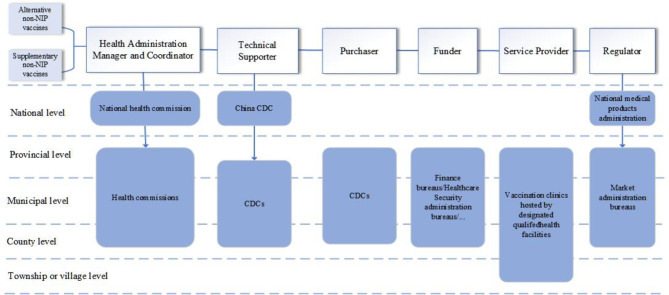
Organization structure of China’s non-NIP vaccines

Both NIP and non-NIP vaccines are inoculated at qualified medical institutions. NIP vaccines are centrally procured and distributed by the national CDC, while non-NIP vaccines are procured by provincial CDCs. NIP vaccines are mainly domestic, whereas non-NIP vaccines include both imported and domestic options. Information on the specific vaccine types, prices, and proportions of imports and domestic production for these two vaccines is provided in Appendix 1–2. Led by the NHC, the management of non-NIP vaccines is conducted through an integrated network. Horizontally, this network includes various stakeholders: health commissions responsible for oversight and coordination; CDCs providing technical support and handling vaccine procurement; vaccine clinics within designated qualified health facilities administering vaccines; funders for financial support and regulators (medical products administrations, China FDA) for compliance. Vertically, governance is organized at multiple administrative governmental levels, with each institution having clear roles and responsibilities. The network is shown in Fig. 1.

### Conceptual framework

Our analysis is anchored on the World Health Organization (WHO) framework, which defines health financing as the function within a health system that involves mobilizing, accumulating, and allocating funds to address both individual and collective health needs [17, 18]. Vaccine financing should be viewed within the broader architecture of healthcare financing. Fund collection involves gathering revenue allocated for vaccines and related service expenses. Pooling and management enable the effective redistribution of resources. Purchasing refers to the mechanisms through which mobilized funds are used to acquire vaccines and pay service providers [19, 20]. To achieve the IA2030 goals of " ensuring good governance, stewardship and accountability of financing for immunization programmes for optimal performance and best value for money“ [21], this can be realized through efforts in three functions.

Given this perspective, a key question arises: How do the design and operation of financing mechanisms, considering the specific context of a country and the unique characteristics of vaccines, influence the fulfillment of these functions? Conversely, how can we optimize these financing functions to enhance public health outcomes through vaccination? Bridging the gap between financing functions and achieving these goals requires a deep understanding of their interconnections. This includes identifying key characteristics and concerns of vaccine financing, such as determining who is covered, which vaccines are included, and what proportion of costs is covered. These concerns are directly linked to the essential features of a good financing mechanism, as outlined by Gavi: it should be equitable (ensuring fair access to vaccines and reducing disparities), effective and efficient (generating and allocating sufficient, timely, and reliable resources in a manner that is not administratively expensive), accountable (providing transparency and responsibility in fund management), and sustainable (maintaining consistent, long-term funding to support current and future immunization targets) [19, 22]. Our analysis will focus on the functions of vaccine financing, its coverage, and performance.

### Study design

A qualitative approach with in-depth, semi-structured interviews was used to gain insights into the decision-makers’ experiences, attitudes, and opinions toward vaccine financing, as well as to assess the feasibility of improving vaccination uptake through potential financing ways. An in-depth interview is a qualitative data collection method that “seeks to foster learning about individual experiences and perspectives on a given set of issues” [23] and acquires detailed information revealing a profound understanding of the research question. To ensure a structured and transparent reporting of our methods and findings, we adhered to the Consolidated Criteria for Reporting Qualitative Research (COREQ) checklist (Appendix 3) [24].

#### Study sites and recruitment

To ensure a comprehensive understanding of vaccine financing in China, we first determined the sample points and selected five provinces from four geographical regions: Guangdong (south), Hubei (central), Sichuan and Yunnan (west), and Liaoning (northeast). In the western region, we added Yunnan as an additional province to gain a more nuanced understanding of the immunization practices within its diverse ethnic minority communities. Factors for choosing these provinces included economic indicators, geographical placement, and local immunization policies. After identifying the sample points, we randomly selected one city and one county within each province, following China’s three-tier administrative system for vaccine uptake. Detailed information on the sampling sites is provided in Appendix 4.

For respondent recruitment, we collaborated with local health authorities to recommend eligible personnel who were familiar with vaccine-related matters and had a minimum of 4 years of professional experience within their departments. To streamline the interview process, we scheduled a unified time for gathering respondents from different departments, including the Health Bureau, CDC, Medical Insurance Bureau, and Finance Bureau. We initially conducted a briefing meeting to explain the purpose and requirements of the interviews. Following this, we carried out one-on-one interviews in offices without any external disruptions to ensure a focused and in-depth discussion.

### Data collection

From July to December 2023, we conducted semi-structured interviews with various stakeholders, and no relationship was established before the interview. The interview protocol was assessed for appropriateness by the research team and three experts working in the field (one health policy researcher expert, and two public health officers).

The interviews were carried out by WJ, MJ, and XY with extensive research experience in the field of health policy, and initiated with a self-introduction for doing the research and open-ended questions, encouraging participants to discuss any issue they deemed relevant to the financing of non-NIP vaccines. Questions were open-ended and included questions such as “What challenges for the department in participating in vaccine financing?”, “How fair do you think the current funding methods are?”, “Do you think it is possible to expand diversified financing channels in China/locally to pay for non-NIP vaccines?”, “What do you think are the reasons for the low coverage of non-NIP vaccines in China?”. To verify that the recommended personnel met the required standards, we began each interview by asking respondents to introduce their work background and experience. We maintained a non-judgmental stance towards any content arising during the interview process, as well as in the subsequent analysis and publication. Interviews lasted between 45 and 60 min with each stakeholder in Mandarin and were conducted until the point of saturation when no new themes emerged.

Interviews were audio-recorded and transcribed verbatim without making field notes and returning them to participants for correction. To ensure confidentiality and anonymity, all data were stored in password-protected files accessible only to the research team. Personal identifiers were omitted from both audio and written transcripts, with numerical identifiers used during analysis (e.g., “1” for the first interview) to maintain participant privacy.

For important explanatory data, such as vaccine prices and government support programs, we conducted searches and gathered information from official platforms and published literature.

### Analysis

All interviews were coded using NVivo 11 software (QSR International Pty Ltd, Victoria, Australia), utilizing a thematic analysis and inductive methodology. The process began with designing the questionnaire based on a well-defined conceptual framework, which guided the formulation of questions to align with our research objectives. The three investigators (i.e., MZ, XY, HM) familiarized themselves with the data to gain a comprehensive understanding and performed initial coding by generating codes directly from the items. Each code was linked to specific themes or concepts from the responses. We then applied these initial codes to analyze the collected data, systematically sorting and examining responses to identify patterns, trends, and key insights related to our research questions. Finally, in the writing-up phase, we organized the analysis results into a table, presenting the relationship between each theme and the associated financing sources, challenges or opportunities. We provide the coding example in Table 1.

**Table 1:** Example of content analysis process

Text	Coding	Emerging Themes	Which source belongs	What represents
Relying solely on out-of-pocket payments cannot meet vaccine demand, especially given the high cost of non-NIP vaccines.	Financial Barriers	Vaccine Affordability	Out-pocket-payment	challenge
Particularly for populations in remote areas, their low income results in greater inequalities in vaccination access.	Geographic Inequality	Access Disparities	Out-pocket-payment	challenge
The current basic medical insurance has great limitations, which cannot be broken through	Insurance Coverage	Insurance Limitations	Insurance	challenge
There are some government support programs to give residents free vaccination	Government Support	Availability of Support Programs	Government fiscal	opportunity
Government support programs depend on local fiscal conditions and require government discussions to determine.	Government Support	Conditional Program Availability	Government fiscal	both
Commercial insurance covers limited vaccines, and the premiums are high.	Insurance Coverage	Insurance Coverage Gaps	Insurance	challenge
The decentralized procurement of vaccines leads to high vaccine prices.	Procurement Challenges	Inefficient Procurement	Out-pocket-payment	challenge

## Results

### Participant characteristics

All interviewees were pre-scheduled, and there was no dropout or duplication of interviews. Finally, we interviewed 55 informed respondents, including 28 females and 27 males. Among them, 16 were from provincial-level departments, with 20 and 19 respondents from city and county levels, respectively. There were 15 participants from the Finance Bureau, 25 from the CDC and Health Bureau, and 15 from the Medical Insurance Bureau.

### Status of current vaccine financing in China

The major non-NIP vaccine financing sources in China are as follows: (1) direct out-of-pocket payments; (2) government fiscal support paid by special funds; (3) the private medical savings account (PMSA) of basic healthcare insurance scheme known as the Urban Employee Basic Medical Insurance (UEBMI) for urban employees and retirees, which is administered by the government; (4) commercial medical insurance provided by private companies; and (5) other external funds. Table 2 presents a summary of the characteristics of these vaccine financing mechanisms based on the interview findings.

**Table 2:** Characteristics of current vaccine financing mechanisms in China

Domain	Resource generation	Pooling and management of funds	Purchasing of health services
Out-of-pocket payment	Individual and household income	Funds are pooled within the household; no central pooling mechanism	Individuals and households make direct payments at the point of accessing immunization care without pre-arranged financial coverage
Government fiscal funds	Specialized funds for immunization are allocated by the government specifically to support programs for public vaccination. The government earmarks these funds to ensure targeted support for immunization initiatives	The allocated funds are pooled into a centralized account managed by the Ministry of Finance. From this pool, budgetary allocations are made to the Ministry of Health. Disbursements are scheduled at regular intervals to ensure continuous support for immunization programs	Vaccines and related services are procured through the Ministry of Health, which is responsible for the implementation of vaccination programs. The costs are co-paid by the Ministry of Finance at various level of government
Basic healthcare insurance	Funds are generated through the contribution of premiums by the insured population. This scheme is designed to be universally inclusive, ensuring broad access to essential healthcare services	Funds are pooled centrally and are typically managed by the health insurance bureau	Purchasing of vaccination services can be done directly from service providers, with payments typically made using the PMSA of the UEBMI
Commercial healthcare insurance	Commercial insurance is offered by private insurance companies. It is also paid by the insured	commercial insurance funds are pooled and managed through private insurance companies	The reimbursement ratio and payment structures for commercial health insurance are determined by the specific terms of the insurance policy, which may vary widely. Policyholders may pay directly for services and then seek reimbursement according to the terms outlined in their commercial insurance plan
Other external funds	Funds from external sources, such as donations and grants, are generated through contributions from individuals, organizations, or philanthropic entities	The external funds are usually pooled into dedicated accounts managed by the recipient organizations, such as non-governmental organizations, charitable foundations, or specific health program offices	Most commonly, direct transfers are employed, facilitating straightforward disbursement of funds to service providers or program implementers

Drawing from the interviews, the coverage scopes across five funding channels are different. Out-of-pocket payments, as the predominant payment method, enable vaccine recipients to select according to residents’ needs, providing the broadest coverage of vaccine types and target populations. An additional vaccination service fees of 15–25 Chinese Yuan (CNY), equivalent to around 2.1–3.6 USD (the exchange rate for USD-CNY = 1:7) are also paid by recipients out-of-pocket. This vaccination service fee amounts to approximately 4.2–7.1% of the average daily expenditure in China (about107 CNY or 15.2 USD). Given that this fee is required for each dose, the total cost will accumulate.

Government-funded programs have specific constraints regarding the types of vaccines (e.g., HPV, influenza, PCV) and target populations. Local government programs provide these vaccines free of charge, and in some areas, they also cover the vaccination service fees. Detailed information on the local program from our interview is shown in Appendix 4. The local governments, after considering factors such as the disease burden, vaccine price, cost-effectiveness, public health priorities, may approve the vaccination initiatives designed to benefit the public. Once approved, the regional health departments take the lead in advancing the program. All relevant stakeholders, including the Departments of Education and Finance, the CDC, the Medical Products Administration, collaborate to promote the free vaccination efforts. To provide a clearer view of government program investments, we have supplemented performance data for the HPV free vaccination program in Guangdong Province in Appendix 5.

China’s UEBMI is financed by PSMA plus a social-risk pooling account (SPA). As of 2023, the number of insured individuals in the UEBMI approximately reached 370 million, with a per capita fund income of nearly 6181.8 CNY (833.1 USD) [25]. Currently, only the PSMA of UEBMI are allowed to pay for non-NIP vaccines in a few provinces. After 2021 the PSMA of UEBMI only included employee contribution, and there is no pooling of funds in fact.

We further summarized participants’ perspectives on the major health financing mechanisms in China across four dimensions: equity, effectiveness and efficiency, accountability, and sustainability, with detailed findings presented in Table 3. In summary, out-of-pocket payment is the least equitable and sustainable, offering immediate but limited access with significant financial barriers. Government fiscal funds provide targeted support with high accountability and potential for equity improvement, but their sustainability is uncertain due to reliance on government input. Basic Healthcare Insurance offers a more equitable and moderately sustainable solution, though it is closely tied to the stability of the insurance system and the scope of its coverage. Each funding source has unique strengths and weaknesses that impact its overall effectiveness in supporting vaccine delivery.

**Table 3:** Summary of coverage and participant views on vaccine financing mechanisms

Domain	Funding Coverage			Respondents’ perceptions of performance			
	Types of Vaccines	Target Populations	Costs	Equity	Effectiveness and efficiency	Accountability	Sustainability
Out of pocket payment	All non-NIP vaccines based on individual demand and availability	General population	Vaccine product fee & vaccination service fee	Inequitable. Access to vaccine is determined by ability to pay	Effective for immediate payment but may result in underutilization due to financial barriers	Individuals handle payments directly with no need for oversight	Uncertain and dependent on individual financial capacity
Government fiscal funds	Specific non-NIP vaccines	Specific groups (vulnerable populations, such as the elderly and school girl)	Vaccine product fee & vaccination service fee (varies in different regions)	Opinions varied. Limited to some areas and beneficiaries, but there is potential for improvement in targeting populations	Efficient because it employs concentrated efforts to achieve high impact	Accountability requirements are strict	Relatively unstable due to depend on government fiscal stability
Basic healthcare insurance	Specific non-NIP vaccines	Specific groups (insured populations)	Vaccine product fee	Relatively equitable	Effective in reducing out-of-pocket costs but requires substantial fund accumulation	Basic insurance has moderate accountability through government oversight	Moderate sustainability
commercial healthcare insurance				Less equitable due to limited coverage	Efficiency is low because of a relatively small pool size and fragmented coverage	Commercial insurance varies with private sector regulations	Relatively stable due to regular premium payments
Other external funds	Specific non-NIP vaccines	Specific groups (vulnerable populations, such as the elderly and female students).	Vaccine product fee	Enhances equity by focusing on remote areas and vulnerable populations	Efficiency is limited by the fragmented nature of the funding pool	Accountability varies; effectiveness depends on the oversight and reporting standards of the managing organizations	Variable sustainability; often project-based and inconsistent

We have summarized and presented the main results of challenges and opportunities in Table 4. Concrete analysis is as follows.

**Table 4:** Challenges and opportunities of non-NIP vaccine financing in China

Domain	Challenges	Opportunities
Out-of-pocket payment	High non-NIP vaccine prices limit residents’ demand for immunization	Increased recognition of the value of immunization and its knowledge among the residents; Changes in market competition have led to price reductions for some vaccines
Fiscal support	The financial inputs lack stability and certainty and even exacerbate coverage inequities	Increased political commitment and more free vaccination programs in economically well-off areas; The centralized purchasing model has been implemented and tested, achieving a “quantity for price” strategy and resulting in substantial price reductions for certain types of non-NIP vaccines; Adopt more diverse forms of support such as subsidies and more options
Health insurance	The Medical insurance-related laws and regulations restrict the involvement of basic health insurance, while the participation of commercial insurance in financing non-NIP vaccines is constrained by the coverage scope and relatively high premiums	Exploring the opening of PMSA in basic health insurance and the establishment of a mutual-aid mechanism for non-NIP vaccines, commercial insurance can meet varied levels of vaccination demand, thereby enhancing the diversity of financing channels
Other funds	No prepaid has been formed and its sustainability is yet to be seen	The involvement of diversified actors (charities, etc.) and simultaneous implementation of health education actions can positively impact vaccination decision-making

### Challenges in existing financing mechanisms

#### Out-of-pocket payment

The primary challenge lies in the high price of non-NIP vaccines and vaccines that rely on a single supplier have higher prices. Such high prices may suppress the demand for non-NIP vaccines within the Chinese market. In appendix 2, we compared the Chinese and international prices of several key non-NIP vaccines.

*“The cost of non-NIP vaccine is expensive. For instance*, *the herpes zoster vaccine can reach 1598 CNY (228 USD) per dose*, *while the nine-valent HPV vaccine is priced at 1298 CNY (185 USD) per dose. Other vaccines*, *the imported 13-valent PCV vaccine*, *and pentavalent vaccines*, *also have high prices. However*, *in the past few years*, *due to the popularization of the COVID-19 vaccines as well as publicity and education*, *residents have become more and more aware of the importance of non-NIP vaccines*, *and many of them*, *although they also think that the price is higher*, *are more receptive*, *or at least no longer resistant to it.“(Interviewee 12 form the provincial health commission)*.

#### Government fiscal support

Regional financial capacity is the most important factor in making decisions for subsidizing public health programs, including vaccination projects. Due to the hierarchical administrative system in China, which operates at the provincial, municipal, and county (district) levels. This structure results in varying population sizes and administrative burdens at each level, with provinces and municipalities generally possessing greater financial resources than districts and counties. Therefore, financially well-off regions can choose to allocate local fiscal resources to cover certain types of non-NIP vaccines. Such local support not only demonstrates a commitment to public health but also contributes to the establishment of herd immunity, thereby reducing the risk of disease transmission within the community and mitigating the economic risks associated with illnesses.

The principal challenges regarding this funding approach are the instability of financial input and the potential inequity. On the one hand, funding for such initiatives is contingent on fiscal capacity, leading to situations where financial support might be offered in one year but reduced or withdrawn in subsequent years if economic conditions worsen. Moreover, as public awareness grows, it requires increasing financial resources to subsidize vaccination projects as more people are receiving the vaccines.

*“Now it’s purely local policy support*, *and the main consideration is whether there is a balance this year. If we took 460*, *000 CNY(65714.2 USD)of support last year and continue to support it this year*, *it may take 900*, *000 CNY (128571.4 USD). If it continues*, *it may be that the people’s knowledge rate will be higher*, *and the pressure on the invested funds will be very troublesome. If there is no balance of money*, *it may not be done. We are most worried about this sustainability.” (Interviewee 7 from the municipal finance bureau)*.

*“Sustainability is difficult to guarantee from the financial sector. For example*, *after this year’s flu vaccination*, *the public may raise questions*, *wondering why they received the influenza vaccine last year but not this year. Moreover*, *people get the free flu vaccine but also want to free PCV*, *so the investment is too big.” (Interviewee 19 from the provincial finance bureau)*.

On the other hand, this approach may lead to regional disparities or a “race to the top” effect. Given China’s transparency in information disclosure, the government could encounter pressures stemming from performance evaluations, potentially prompting other regions to consider increased investments in similar fiscal programs. While this mechanism improves vaccination in economically-developed cities in general, regions with limited financial resources lagged behind.

*“There will be a catching-up effect between cities. For example*, *if one city initiates free influenza vaccinations*, *another neighboring city may feel stressed and consider adopting a similar vaccination program. If one city continues this program for two consecutive years*, *another may have had to schedule a program like this.” (Interviewee 13 from the provincial finance bureau)*.

#### Basic health insurance

Currently, the basic medical insurance-related laws and regulations are the main constraints for its participation in the financing of non-NIP vaccines. Vaccines are categorized as preventative services within the domain of public health and thus fall outside the payment scope of the basic medical insurance pooled funds. Furthermore, for PMSA of the UEBMI, rigorous management regulations are in place, precluding the use of these accounts for vaccination in most areas.

*“Currently*, *the basic health insurance cannot cover expenses that should be borne by public health according to China’s authoritative laws. Vaccines are generally considered to be within the domain of public health. The original purpose of basic medical insurance was to protect essential healthcare*, *the social insurance law was passed by the People’s Congress*, *we must keep the law.” (Interviewee 5 from the provincial medical insurance bureau)*.

Regarding the process of payments, the fundamental criteria for health insurance coverage are encapsulated within three key catalogs: drugs, diagnostic and therapeutic items, and medical service facilities. Services or products absent from these catalogs do not qualify for reimbursement through the basic healthcare insurance fund. Current regulations stipulate that vaccine is not included in the above catalog, thereby rendering them ineligible for coverage.

*“The benchmark for health insurance payment is the three major catalogs that I have introduced*, *and if this thing is not in the three major catalogs*, *the medical insurance funds can’t pay for it according to the regulations. “(Interviewee 41 county from the medical insurance bureau)*.

*“At present*, *all matters about medical insurance are centralized under national governance*, *with no authority vested in the provinces and municipalities. we can only follow the national policy to implement.” (Interviewee 5 from the provincial medical insurance bureau)*.

In addition to the difficulty of revising the legal provisions in the short term, managers of the health insurance fund also prioritize healthcare treatment security not investing in preventive services when there is a surplus of funds.

*“From an individual perspective*, *the main contradiction needs to be captured. The priority need is to ensure outpatient*, *inpatient*, *and emergency patients are protected*, *in the case of the security and sustained of the fund*, *we can continue to improve the level of reimbursement to reduce the burden of patients’ medical care. The incorporation of preventive measures into medical insurance coverage may represent the next stage. However*, *we must first adequately fulfill the fundamental requirements before contemplating the subsequent phase.” (Interviewee 35 from the municipal medical insurance bureau)*.

#### Commercial medical insurance

Interviewees pointed out that the current involvement of commercial insurance in non-NIP vaccines in China is insufficient. In the private market, commercial insurance primarily focuses on fundamental healthcare needs such as major illnesses, cancer, inpatient and outpatient services, with a limited selection of products offering reimbursement for vaccines.

*“Whether commercial insurance pays for vaccines depends on the premium and specific terms. Most of the commercial insurance covers some major illnesses and some supplemental reimbursement outside the basic health insurance catalog.” (Interviewee 7 from the provincial medical insurance bureau)*.

Furthermore, the comprehensive commercial insurance plans that reimburse vaccinations usually have higher premiums and need to consider its affordability. These insurance plans may be restricted to covering specific types of vaccines and set annual limits within each insurance period.

*“There are very few commercial insurances on the market that have reimbursement for vaccines*, *and even if there are*, *the insurance premium is high. Most of our people still rely on the basic health insurance.” (Interviewee 33 from the municipal medical insurance bureau)*.

### Opportunities within current financing mechanisms

#### Out-of-pocket payment

Notably, in recent years, the population’s awareness and acceptance for non-NIP vaccines has increased, leading to higher demand for vaccination. Due to a decline in the number of newborns and advancements in vaccine technology for domestic companies, the market non-NIP vaccines in China are becoming increasingly competitive. To capture a larger market share, some domestic companies have reduced their vaccine prices. For instance, China National Pharmaceutical Group (Sinopharm) lowered the price of its influenza vaccine to below 100 CNY (14.2 USD) in some areas, prompting other domestic companies such as Sinovac, Hualan Biological, and CanSino to also decrease the prices of their quadrivalent influenza vaccines. Lower prices increase the affordability of vaccines, thereby potentially improving the equity in vaccine coverage across regions. Nevertheless, inequity in the financing for non-NIP vaccines still exists with out-of-pocket payments as the major approach.

#### Basic healthcare insurance

The most significant policy change is that the immediate family members, including spouses, parents and children, will also be able to use the PMSA of insured employees for medical bills at designated institutions, namely “the mutual-aid mechanism”. Traditionally, the population covered by the UEBMI are those who are currently employed or retired workers, excluding children and adolescents. It has also been observed that individual account balances are concentrated among the younger population, the elderly has comparatively lower account surplus rates and amounts. Besides, children are the main group receiving non-NIP vaccines, but they have no insurance to cover the cost. Now after the implementation of the mutual-aid mechanism, some regions such as Nanjing and Suzhou have made adjustments accordingly, allowing insured individuals to use the PMSA for non-NIP vaccine payments for themselves and their family members. The mutual assistance and co-payment system within families facilitates the effective utilization of surplus funds to meet the vaccination needs of family members.

#### Fiscal support

In vaccination programs with fiscal support, as the targets for vaccination are clearly defined, including the age, gender, and residency requirements, the purchase quantities can be clearly predicted. Besides, with increasing vaccination campaign organized by the government, the projected demand for vaccinations is expected to rapidly increase, thereby leading to a substantial scale of procurement. To succeed in competitive bidding for such a large market, companies have chosen to reduce prices. For example, Guangdong province procured more than 1.3 million doses of bivalent HPV vaccine for middle school girls aged 12–14 to be vaccinated in 2023. The price of vaccines from the same domestic manufacturer has decreased from a self-pay market selling price of 339 CNY (48.4 USD) to 116 CNY (16.5 USD) per dose, a reduction of nearly 65%. Similar successful negotiations on prices in free vaccination programs have occurred in other regions, such as PCV and influenza vaccines. These practices prove that e centralized procurement with large quantities and competitive bidding rules help to achieve efficient procurement of non-NIP vaccines.

*“During direct procurement*, *non-NIP vaccines generally maintain a uniform price nationwide*, *with minimal room for price reduction. When negotiating*, *manufacturers said that if a province reduces its price*, *it could trigger a nationwide chain reaction*, *causing price fluctuations across the country. Consequently*, *achieving price reductions becomes challenging.” (Interviewee 27 from the provincial medical insurance bureau)*.

*“I heard that Guangdong*, *Jiangsu Province were able to negotiate lower prices for PCV vaccines and other vaccines when implementing their regional free immunization programs*, *indicating that there is still room for price negotiation. Maybe we can absorb the experience of centralized drug procurement.” (Interviewee 23 from the provincial medical insurance bureau)*.

Some regions chose to offer government subsidies for the vaccination of certain vaccines, instead of organizing vaccine procurement. This strategy can also incentivize the target population to receive the vaccines. For instance, in Sichuan Province, a fixed subsidy of 600 CNY (85.7 USD) per person is granted for the administration of the bivalent and quadrivalent HPV vaccines to eligible girls aged 13–14. Nanjing City offers the government’s directly procured bivalent HPV vaccine free of charge, while providing a fixed subsidy of 492 CNY (70.2 USD) for other valent HPV vaccines.

*“Some regions also support through subsidies*, *which can not only play the protective role of vaccines*, *but also reduce financial pressure to a certain extent. Chengdu City set a subsidy of 600 CNY (85.7 USD)per individual for HPV vaccination for students aged 13–14 years old*, *and the subsidy is shared by the city*, *county*, *and district finances. Mianyang City also adopts a similar policy.” (Interviewee 11from the municipal medical insurance bureau)*.

#### Other funds

Organizations such as charitable associations, hospitals, and women’s federations have initiated various philanthropic initiatives. The contributors have borne the cost of vaccines, encouraged more people to receive the HPV vaccines and conducted promotional and educational initiatives, which has increased the community’s awareness.

## Discussion

To our knowledge, this qualitative study is the first to comprehensively examine the practice and progress of non-NIP vaccine financing in China. Our findings reveal that China’s current heavy reliance on out-of-pocket payments for non-NIP vaccine financing fails to optimize the benefit of vaccines and is inequitable. The latest comparative data from 2021 revealed that the prices of non-NIP vaccines from both domestic and foreign manufacturers in China were much higher than those set by UNICEF, but similar to or even higher than the prices charged for the CDC and private sector in the USA [14]. This price disparity brings the financial burden on individuals. While other funding channels, including government fiscal support, insurance, and external funding, do contribute, they each face legal restrictions or sustainability issues and operate in isolation. Additionally, the coverage provided by basic health insurance and commercial insurance remains limited.

This study also identifies several opportunities for improving financing schemes for non-NIP vaccines. The permission of PSMA of UEBMI to pay for non-NIP vaccines, and the establishment of mutual aid mechanisms which enables family members to use PSMA are promising approaches. Furthermore, the substantial reduction in vaccine prices in some programs receiving fiscal support validates the feasibility of concentrated procurement. This further implies that changes in the financing approach can reshape the procurement strategies to improve the bargaining power in price negotiation, and consequently reaching lower prices. Based on these insights, we propose strategies into two key areas as outlined in IA20230: (1) ensuring sufficient, predictable resources; and (2) improving public financial management.

In the first area, on the one hand, it is crucial to advocate for and design financing strategies at the national level that prioritize universal coverage for vaccines with robust evidence of benefits. Robust economic evaluations, such as Financial Value of Vaccine Assessments (FVVA), offer a comprehensive approach that considers factors like demographics, healthcare capacity, and competing priorities, and can be used to support government decision-making [26]. On the other hand, diversifying funding sources is vital, with the potential to deepen the involvement of local finance and health insurance. For local finance, recent studies have evaluated three funding strategies for influenza vaccine and showed that a multifaceted cost-sharing financing model involving individuals, central, and local finances can mitigate economic burdens among diverse stakeholders and enhance health performance [27]. In 2018, China established five-tier expenditure responsibility standards for essential public health programs, defining the distribution of financing between the central and provincial governments. These standards delineate five different expenditure ratios for essential public health services, with the central government assuming different proportions of the financial burden based on the different socio-economic circumstances in the provinces [28]. Similarly, a cost-sharing mechanism can be developed between the central and local finance for vaccine initiatives, based on a careful examination of local fiscal capacities [29]. The cost-effectiveness of financial investment is recommended to support evidence-based decision-making. Additionally, it is advised to consider gradually pushing forward the inclusion of non-NIP vaccines in the NIP to allow for dynamic vaccine adjustments [13].

Regarding health insurance, vaccines are covered by various government and private insurance programs in many countries. For example, the USA mandate that insurance plans cover all CDC-recommended vaccines [30]. Considering that vaccination may greatly reduce future healthcare expenditures, save the medical insurance fund, and improve its efficiency, it may be beneficial to increase the discussion on existing legislation. With reference to the current reimbursement policy of drugs, varying reimbursement rates can also be set for different vaccines. Besides, currently China is actively promoting mutual-aid policy within family members for PMSA and expanding the payment scope of PMSA to include selected non-NIP vaccines might be advantageous.

In the second area, the focus should be on: first, to improve vaccine demand forecasting and budgeting; and second, to establish innovation financing model. Centralized procurement with guaranteed quantities has proven to be a feasible approach for demand forecasting, budgeting, and procurement. Some local fiscal programs have demonstrated that substantial and stable quantities can be utilized in price negotiation to reduce vaccine prices. Globally, pooled procurement is viewed as an effective demand-side strategy to address supply chain risks in vaccine markets, such as dispersed demand, high per-unit product costs, inaccurate or delayed forecasting, and supply planning [31, 32]. It is typically performed within supranational entities (e.g., Pan American Health Organization, UNICEF, Vaccine Alliance, Gulf Cooperation Council Group Purchasing Program [33, 34] and receives the benefits through consolidating forecasted demand requirements and leveraging economies of scale [35, 36]. The vaccine purchasing group in the USA obtains discounted vaccine pricing based on the product loyalty benchmark, which means a certain proportion of the manufacturer’s product line [37]. Another illustrative example is advance market commitments (AMC) of PCVs in low- and middle-income countries (LMICs). The high cost and limited availability of PCVs restrict their use in resource-constrained settings. To address this, Gavi and the WHO implemented the AMC in 2009, which ensured a guaranteed volume of doses at reduced prices. This arrangement provided financial stability for manufacturers, enabling them to increase production and develop distribution networks. Additionally, Gavi effectively utilized resources from both public and private donors to negotiate favorable terms [38].

### Limitations

The findings of this study should be interpreted with consideration of some important limitations related to the study design and settings. Firstly, due to budget and time constraints, we used a sampling method to select 5 provinces for research. While there is a certain level of consistency in the management of non-NIP vaccines across China, we further supplemented our findings with literature and news reports from various regions, the study’s generalizability may still be limited. Secondly, we did not include broader perspectives (e.g., vaccine manufacturers, caregivers). Their involvement in vaccine financing and potential insights were not taken into account. Finally, for privacy reasons, we will only report the gender and department of the interviewees. Furthermore, by employing a qualitative approach, the findings provide a foundational understanding of non-NIP vaccine financing in China. However, the lack of publicly available and accessible financing data highlights the need for future studies to incorporate mixed research methods to capture a more comprehensive picture.

## Conclusions

Ideal financing arrangements for healthcare service should generate adequate, timely, and reliable resource funds and create more benefit packages with lower administrative expenses. The current financing mechanism for non-NIP vaccines in China heavily relies on out-of-pocket payment, which fails to optimize the benefit of vaccines and induces inequity in vaccine coverage. Therefore, it is crucially important to develop and implement concerted actions and synergistic approach, focusing on diversifying funding channels, innovating financing methods and improving the management of public funding for non-NIP vaccines.

## Electronic supplementary material

Below is the link to the electronic supplementary material.

Supplementary Material 1

## Data Availability

No datasets were generated or analysed during the current study.

## Abbreviations

CDC: Centers for Disease Control and Prevention
COREQ: The Consolidated Criteria for Reporting Qualitative Research
CNY: Chinese Yuan
Gavi: Vaccine Alliance
HPV: Human Papillomavirus Vaccine
IA2030: Immunization Agenda 2030
NIP: National Immunization Program
Non-NIP: Non-National Immunization Program
NHC: National Health Commission
PCV: Pneumococcal Conjugate Vaccine
PMSA: Private medical savings account
SDGs: Sustainable Development Goals
UHC: Universal Health Coverage
UEBMI: The urban employee basic medical insurance
UNICEF: United Nations International Children’s Emergency Fund
USA: The United States
UK: The United Kingdom
USD: United States Dollars
WHO: World Health Organization
