# Supplementary material for: The equity road ahead for financing non-national immunization program vaccines in China: challenges and opportunities from a qualitative study
Source: Int J Equity Health. 2024 Sep 27;23:193. doi: 10.1186/s12939-024-02282-5 (PMC11429117; doi:10.1186/s12939-024-02282-5)
Supplement: Supplementary file 1 — Supplementary Material 1 [file 12939_2024_2282_MOESM1_ESM.docx]

**Appendix 1: The catalog of NIP and non-NIP vaccines in China**

| NIP vaccines | - Bacillus Calmette-Guérin vaccine (BCG) - Poliovirus vaccine - Hepatitis B vaccine(HepB) - Diphtheria, pertussis, and tetanus vaccine (DPT) - Diphtheria and tetanus vaccine (DT) - Measles vaccine - Measles, mumps, and rubella vaccine(MMR) - Group A meningococcal polysaccharide vaccine(MPV-A) - Group A and group C meningococcal polysaccharide vaccine(MPV-AC) - Japanese encephalitis vaccine(JE) - Hepatitis A vaccine（HepA） - Hemorrhagic fever vaccine(HFV） - Anthrax vaccine - Leptospirosis vaccine |
| --- | --- |
| Non-NIP vaccines | |
| - Supplementary non-NIP vaccines | - Rabies Vaccine (RV) - Enterovirus Type 71 Vaccine (EV71) - Varicella Vaccine (VAR) - Trivalent Influenza Vaccine (TIV) - Quadrivalent Influenza Vaccine (QIV) - Haemophilus Influenzae Type b Conjugate Vaccine (Hib) - 13-valent Pneumococcal Polysaccharide Conjugate Vaccine (PCV13) - 23-valent Pneumococcal Polysaccharide Vaccine (PPSV23) - Oral Rotavirus Live Vaccine (ORV) - Trivalent rotavirus vaccine (TRV) - Pentavalent Rotavirus Live Vaccine (PRV) - Bivalent Human Papillomavirus Vaccine (2vHPV) - Quadrivalent Human Papillomavirus Vaccine (4vHPV) - 9-valent Human Papillomavirus Vaccine (9vHPV) - Cholera Vaccine (CV) - Tick-Borne Encephalitis Vaccine (TBEV) - Hepatitis E Vaccine (HEV) - Typhoid Vaccine (TyphV) - Plague Vaccine (PV) - Brucellosis Vaccine (BV) - Yellow fever Vaccine(YF) - Herpes zoster vaccine(HZV) |
| - Alternative non-NIP vaccines | - Group A and group C meningococcal polysaccharide conjugate vaccine(MPCV-AC) - Groups A,C,Y and W meningococcal Polysaccharide vaccine（MPV-ACYW4） - HepatitisA vaccine(HepA) - HepatitisB Vaccine(HepB) - Hepatitis A and B Combined Vaccine(HepAB) - Tetanus Vaccine(TV) - Mumps Vaccine(MuV) - Rubella Vaccine(RV) - Group A and C meningococcal polysaccharide conjugate and Haemophilus influenzae type b conjugate combined vaccine (MPCV-AC-Hib) - Diphtheria, tetanus, and pertussis combined with haemophilus influenzae Type b Vaccine (DTaP-Hib) - Diphtheria and tetanus toxoids and acellular pertussis adsorbed, inactivated poliovirus and haemophilus B conjugate vaccine (DTaP-IPV-Hib) |

**Appendix 2：The information of price and supplier of key non-NIP vaccines in China**

| Vaccine Type | Vaccine valence | Number of manufactures  in China | | Price range in China/Dose | US CDC Cost/ Dose | US Private Sector Cost/ Dose | PAHO price/Dose |
| --- | --- | --- | --- | --- | --- | --- | --- |
|  |  | Domestic manufacturer | Import manufacturer |  |  |  |  |
| Human Papillomavirus Vaccine | Bivalent | 2 | 1 | $47.0-84.3 | / | / | / |
|  | Quadrivalent | 0 | 1 | $115.7 | / | / | $10.4 |
|  | 9-valent | 0 | 1 | $187.1 | $240.3 | $287.5 | / |
| Rotavirus vaccine | Monovalent | 1 | 0 | $21.9 | $108.4 | $138.7 | $6.5 |
|  | Trivalent | 1 | 0 | $33.3 | / | / |  |
|  | Pentavalent | 0 | 1 | $41.9 | $81.5 | $95.9 |  |
| Pneumococcal vaccine | 23-valent | 4 | 1 | $28.1-44.0 | / | / | / |
|  | 13-valent | 2 | 1 | $67.6-101.1 | $65.8 | $117 | $12.9 |
| Influenza Vaccine | Trivalent | 8 | 1 | $7.9-20.4 | $15.07-21.4 | $19.7-32.4 | $1.3-3.7 |
|  | Quadrivalent | 7 | 1 | $14.0-30.4 |  |  | $4.5-5.3 |
| Hib Vaccine | / | 5 | 1 | $12.6-17.6 | $11.0-16.1 | $12.8-29.7 | $2.2 |
| Herpes zoster vaccine | / | 1 | 1 | $197.7-229.7 | / | / | / |

Source：

1.List of non-immunization program vaccines used in Guangdong Province in 2024（<https://www.gdmede.com.cn/announcement/announcement/detail?id=1800419788804067328>.

We used the 2024 price data for Guangdong Province, mainly because there is currently no national price data in China. Additionally, Guangdong requires that the sales prices of non-NIP vaccines distributed within the province not be significantly higher than the national average prices during the same period and remain relatively stable. The conversion was made using a 1:7 exchange rate.

2.USA CDC Vaccine Price List(<https://www.cdc.gov/vaccines-for-children/php/awardees/current-cdc-vaccine-price-list.html>)

3.PAHO Revolving Fund Vaccine Prices for 2023(https://www.paho.org/en/documents/paho-revolving-fund-vaccine-prices-2023)

**Appendix 3: COREQ (Consolidated criteria for Reporting Qualitative research) Checklist**

| Topic | Item No. | Guide Questions/Description | Reported on Page No. |
| --- | --- | --- | --- |
| Domain 1:Research team and reflexivity | | | |
| Personal characteristics | | | |
| Interviewer/facilitator | 1 | Which author/s conducted the interview or focus group? | 5 |
| Credentials | 2 | What were the researcher’s credentials? e.g. PhD, MD | NA |
| Occupation | 3 | What was their occupation at the time of the study? | 4 |
| Gender | 4 | Was the researcher male or female? | 6 |
| Experience and training | 5 | What experience or training did the researcher have? | 5 |
| Relationship with participants | | | |
| Relationship established | 6 | Was a relationship established prior to study commencement? | 5 |
| Participant knowledge of the interviewer | 7 | What did the participants know about the researcher? e.g. personal goals, reasons for doing the research | 5 |
| Interviewer characteristics | 8 | What characteristics were reported about the interviewer/facilitator? e.g. Bias, assumptions, reasons and interests in the research topic | 5 |
| Domain 2:Study design | | | |
| Theoretical framework | | | |
| Methodological orientation and Theory | 9 | What methodological orientation was stated to underpin the study? e.g. grounded theory, discourse analysis, ethnography, phenomenology, content analysis? | 5 |
| Sampling | 10 | How were participants selected? e.g. purposive, convenience, consecutive, snowball | 4 |
| Method of approach | 11 | How were participants approached? e.g. face-to-face, telephone, mail, email | 5 |
| Sample size | 12 | How many participants were in the study? | 6 |
| Non-participation | 13 | How many people refused to participate or dropped out? Reasons? | 6 |
| Setting | | | |
| Setting of data collection | 14 | Where was the data collected? e.g. home, clinic, workplace | 5 |
| Presence of non-participants | 15 | Was anyone else present besides the participants and researchers? | 5 |
| Description of sample | 16 | What are the important characteristics of the sample? e.g. demographic data, date | 6 |
| Data collection | | | |
| Interview guide | 17 | Were questions, prompts, guides provided by the authors? Was it pilot tested? | 5 |
| Repeat interviews | 18 | Were repeat interviews carried out? If yes, how many? | 6 |
| Audio/visual recording | 19 | Did the research use audio or visual recording to collect the data? | 5 |
| Field notes | 20 | Were ﬁeld notes made during and/or after the interview or focus group? | 5 |
| Duration | 21 | What was the duration of the interviews or focus group? | 5 |
| Data saturation | 22 | Was data saturation discussed? |  |
| Transcripts returned | 23 | Were transcripts returned to participants for comment and/or correction? | 5 |
| Domain 3: analysis and analysis | | | |
| Data analysis | | | |
| Number of data coders | 24 | How many data coders coded the data? | 5 |
| Description of the coding tree | 25 | Did authors provide a description of the coding tree? | 5 |
| Derivation of themes | 26 | Were themes identiﬁed in advance or derived from the data? | 5 |
| Software | 27 | What software, if applicable, was used to manage the data? | 5 |
| Participant checking | 28 | Did participants provide feedback on the ﬁndings? | 5 |
| Reporting | | | |
| Quotations presented | 29 | Were participant quotations presented to illustrate the themes/ﬁndings? Was each quotation identiﬁed?e.g. participant number | 6-12 |
| Data and ﬁndings consistent | 30 | Was there consistency between the data presented and the ﬁndings? | 6-12 |
| Clarity of major themes | 31 | Were major themes clearly presented in the ﬁndings? | 6-12 |
| Clarity of minor themes | 32 | Is there a description of diverse cases or discussion of minor themes? | 6-12 |

Reference:

Tong, A., Sainsbury, P., & Craig, J. (2007). Consolidated criteria for reporting qualitative research (COREQ): a 32-item checklist for interviews and focus groups. International journal for quality in health care : journal of the International Society for Quality in Health Care, 19(6), 349–357.

**Appendix 4: Basic information of sample sites**

| Province | Population (in ten thousand) | Regional Gross Domestic Product  (in billion CNY) | Public budget revenue (in billion CNY) | Per capita disposable income  (CNY) | Is there vaccine programs that funded by local fiscal? | Programs Details | Vaccination service fee (per dose/ CNY) |
| --- | --- | --- | --- | --- | --- | --- | --- |
| Liaoning | 4197 | 28975.1 | 2525.07 | 36088.8 | Yes, not continuously ongoing | In 2021, Dalian city offered a free dose of influenza vaccine to all citywide residents aged 60 and above | 23 |
| Yunnan | 4693 | 28954.2 | 1949.46 | 26936.8 | Yes, partially ongoing | From 2018 to 2020, Kunming city offered free vaccination of the 23-valent pneumococcal vaccine for individuals aged 60 and above  From January 1, 2023 to December 31, 2025, Yuxi city provides free 2-valent HPV vaccination for female students in the first year of middle school | 15 |
| Sichuan | 8374 | 56749.8 | 4880.55 | 30679.2 | Yes, still ongoing | From 2021 to 2025, school girls aged 13-14 years old in Chengdu City can independently choose between domestic/imported bivalent and imported quadrivalent HPV vaccines, and are given a subsidy of 600 yuan per person | 20 |
| Guangdong | 12657 | 129118.6 | 13260.88 | 47064.6 | Yes, still ongoing | From September 2022, domestic 2-valent HPV vaccine will be administered to female students under the age of 14 who have Guangdong provincial school registration and have not received the HPV vaccine upon entering the first year of junior high school | 21 |
| Hubei | 5844 | 53734.9 | 3281.13 | 32913.6 | No | / | 20 |

*The above regional information is from the China Statistical Yearbook 2023, while the vaccine-related information is derived from interviews and official government websites.

**Appendix 5: Costs on the implementation of government-funded projects-a case of Guangdong Province**

- Implement period: September 2022-present
- Target population: Girls under the age of 14 who have a school record in Guangdong Province (covering 21 cities) and have not been vaccinated against HPV since September 2023 will be offered bivalent HPV vaccination.
- Implementation effect: Over 1.28 million seventh-grade girls from the 2022 and 2023 grades have received the HPV vaccine, with vaccination rates exceeding 80%.
- Capital source: The provincial-level and municipal-level, county-level finances jointly bear the costs and distribute them according to a specified proportion
- Capital use: Project funds are allocated to various activities, including providing supplier subsidies for procurement agency fees, information system maintenance, public awareness campaigns, research and evaluation, educational outreach, personnel training, project quality control, supervision, and vaccination cost based on capital standard.

| **Year** | **2022** | **2023** | **From January to March 2024** |
| --- | --- | --- | --- |
| Capital standard (CNY) | 728 CNY/per person：(Vaccine product fee-339 CNY/dose+ vaccination service fee-25 CNY/dose) ×2 dose | 700 CNY/per person：(Vaccine product fee-329 CNY/dose+ vaccination service fee-21 CNY/dose) ×2 dose | 274 CNY/per person：(Vaccine product fee-116CNY/dose+ vaccination service fee-21 CNY/dose) ×2 dose |
| Budget (ten thousand CNY) | 17841.34 | 23361 | 20203 |
| Actual expenditure (ten thousand CNY) | 15067.05 | 10559.41 | 132.2 |

Source：A self-evaluation report on the key performance evaluation of provincial financial funds for the free HPV vaccine vaccination project for school-age girls in 2022-2024(https://wsjkw.gd.gov.cn/attachment/0/555/555549/4448378.pdf)
